# Effect of oral cinnamon intervention on metabolic profile and body composition of Asian Indians with metabolic syndrome: a randomized double -blind control trial

**DOI:** 10.1186/s12944-017-0504-8

**Published:** 2017-06-12

**Authors:** Sonal Gupta Jain, Seema Puri, Anoop Misra, Seema Gulati, Kalaivani Mani

**Affiliations:** 1 Department of Foods and Nutrition, Institute of Home Economics, University of Delhi, New Delhi, India; 2 Fortis-C-DOC Centre of Excellence for Diabetes, Metabolic Diseases and Endocrinology, B-16, Chirag Enclave, New Delhi, -110048 India; 3 Centre of Nutrition & Metabolic Research (C-NET), National Diabetes, Obesity and Cholesterol Foundation (N-DOC), SDA, New Delhi, India; 4 Diabetes Foundation (India), SDA, New Delhi, India; 5 Department of Biostatistics, All India Institute of Medical Sciences, New Delhi, India

**Keywords:** Metabolic syndrome, Cinnamon, Fasting blood glucose, Glycosylated haemoglobin, Blood pressure, Body composition

## Abstract

**Background:**

Nutritional modulation remains central to the management of metabolic syndrome. Intervention with cinnamon in individuals with metabolic syndrome remains sparsely researched.

**Methods:**

We investigated the effect of oral cinnamon consumption on body composition and metabolic parameters of Asian Indians with metabolic syndrome. In this 16-week double blind randomized control trial, 116 individuals with metabolic syndrome were randomized to two dietary intervention groups, cinnamon [6 capsules (3 g) daily] or wheat flour [6 capsules (2.5 g) daily]. Body composition, blood pressure and metabolic parameters were assessed.

**Results:**

Significantly greater decrease [difference between means, (95% CI)] in fasting blood glucose (mmol/L) [0.3 (0.2, 0.5) *p* = 0.001], glycosylated haemoglobin (mmol/mol) [2.6 (0.4, 4.9) *p* = 0.023], waist circumference (cm) [4.8 (1.9, 7.7) *p* = 0.002] and body mass index (kg/m2 ) [1.3 (0.9, 1.5) *p* = 0.001] was observed in the cinnamon group compared to placebo group. Other parameters which showed significantly greater improvement were: waist-hip ratio, blood pressure, serum total cholesterol, low-density lipoprotein cholesterol, serum triglycerides, and high-density lipoprotein cholesterol. Prevalence of defined metabolic syndrome was significantly reduced in the intervention group (34.5%) vs. the placebo group (5.2%).

**Conclusion:**

A single supplement intervention with 3 g cinnamon for 16 weeks resulted in significant improvements in all components of metabolic syndrome in a sample of Asian Indians in north India.

**Trial registration:**

The clinical trial was retrospectively registered (after the recruitment of the participants) in ClinicalTrial.gov under the identification number: NCT02455778 on 25th May 2015.

## Background

Developing countries, particularly in South Asia, are witnessing a rapid increase in prevalence of type 2 diabetes (T2DM) and cardiovascular disease (CVD). During the previous three decades, the prevalence of T2DM has doubled in India, and early onset and severe CVD is frequently seen [1].The reasons for such rapid rise are multiple; but mostly related to imbalanced diets and increased physical inactivity due to economic liberalization, urbanization and mechanisation. In addition, it is the tendency of Asian Indians to have excess body fat, ectopic fat and a resultant high level of insulin resistance. It is not clear if yet unknown genetic tendency or innate defect of metabolism is fueling such a dysmetabolic state [2].

Metabolic syndrome comprises of a clinical state in which abdominal obesity, impaired glucose tolerance, atherogenic dyslipidemia [high serum triglycerides and low high density lipoprotein-cholesterol (HDL-C) levels], hypertension as well as pro-thrombotic and pro-inflammatory states cluster together in the same individual [3]. Individuals with metabolic syndrome are at a five times greater risk of developing T2DM and three times more likely to have a heart attack or stroke compared to people without it [4]. These individuals are also twice as more susceptible to die from T2DM and heart attack or stroke [4]. Almost 20–30% of the population in urban cities of India has metabolic syndrome [5].

It is important to intervene in metabolic syndrome in order to prevent T2DM and CVD. Nutritional modulation and physical activity remain central to any such intervention. It is important to note that some nutraceuticals or functional foods have been shown to decrease atherosclerosis [6]. For example, the proanthocyanidins, major polyphenols in black grape seed, have been demonstrated to have lipid-lowering effects [7]. Plant sterols have been shown to improve lipid profile and have been used in clinical practice [8]). Several other natural functional remedies like alpha lipoic acid [9], biotin [10], pycnogenol [11], and silymarin [12] have been previously investigated, but remain largely inconclusive.

Cinnamon bark (*Dalchini* in Hindi), known from ancient times in the Mediterranean region, Sri Lanka and India, has been used for cooking traditional Indian, Turkish and Persian cuisines, to provide flavour to curries and other food items. The major components present in cinnamon include cinnamaldehyde, cinnamic acid, eugenol, and coumarin [13]. However, the water-soluble polyphenol compounds present in cinnamon which display insulin-potentiating, antioxidant, and related activities are Type A doubly linked procyanidin oligomers of the catechins and epicatechin. Further, methyl chalcone polymer in cinnamon enhances the triacylglycerol lipase activity that hydrolyzes dietary fat molecules, increases glycogen synthesis in liver, enhances glucose uptake and phosphorylation of insulin receptor in skeletal muscles and adipocytes [14].

Some previous studies suggest a potential role of cinnamon and its components in improving insulin sensitivity [15, 16], reducing fasting blood glucose (FBG) [17–20], postprandial blood glucose levels (PPG) (2 h post breakfast) [21, 22], glycosylated haemoglobin (HbA1C) [23], total cholesterol [18], serum triglycerides [18], low density lipoprotein-cholesterol (LDL-c) [18], blood antioxidant levels [19], systolic blood pressure (SBP) [19] and percent body fat [19]. However, among the few human trials conducted, only one which has been conducted on 22 individuals with prediabetes and metabolic syndrome has shown significant decrease in hyperglycemia, blood pressure and body composition parameters [19].

Asian Indians have long been considered to be a “high-risk population” for metabolic syndrome, T2DM and CVD [24]. Appropriate cost effective and culturally suitable diet-based interventions are essential to prevent T2DM and CVD. Hence, this 16 week double blind randomized control study aimed to evaluate the use of cinnamon as a dietary intervention in individuals with metabolic syndrome. We hypothesized that the use of cinnamon would ameliorate risk factors associated with the metabolic syndrome.

## Methods

### Study design

This randomized, double-blind placebo controlled trial of oral cinnamon supplementation on individuals with metabolic syndrome was carried out for 16 weeks after an initial run in period of 4 weeks. The clinical trial was registered after the recruitment of the participants in ClinicalTrial.gov under the identification number: NCT02455778 (URL:https://clinicaltrials.gov/show/NCT02455778?displayxml=true).

### Participants

Subjects were enrolled in the study from October 2011 to April 2012. The last subject completed the study in September 2012. Individuals with metabolic syndrome were identified and recruited from a private hospital and a clinic, located in South Delhi. The Modified NCEP ATPIII criteria recommended for Asian Indians was used for identifying individuals with metabolic syndrome included the presence of at least three of the five components [25]. These include abdominal obesity [waist circumference (WC; men >90 cm; women >80 cm)], high serum triglycerides (TGs ≥150 mg/dL), low HDL-C (men <40 mg/dL; women <50 mg/dL), dysglycemia (FBG ≥100 mg/dL) and hypertension (≥130/≥ 85 mmHg) [25]. All the subjects were newly detected and treatment naïve. Those suffering from uncontrolled hypertension (an average SBP ≥140 mmHg or DBP ≥90 mmHg), severe hypertriglyceridemia (serum triglycerides >400 mg/dL) and hypothyroidism/ hyperthyroidism were excluded. Subjects suffering from other chronic diseases and metabolic complications such as CVD, diabetes, renal disease, myocardial infarction and other endocrinal disorders, any debilitating disease such as tuberculosis, HIV etc. or those on medication of lipid lowering or hypoglycemic drugs were also excluded. Also, only subjects who were stable, if on medication for high blood pressure with no change in dosage over the past 3 months were included in the study.

Participants were also screened for any reported wheat allergy since the placebo consisted of capsules containing whole wheat flour. The study protocol was approved by Institutional Ethics Committees of Fortis Hospital on 6th September 2011 and Institute of Home Economics on 6th September 2011. The patient information sheet was explained to the subjects by the investigator prior to the study which provided information on the purpose of the study, procedures involved, possible risk(s), confidentiality and rights of the subjects. Written informed consent was obtained from all the subjects.

### Randomization

The subjects were allocated to either cinnamon intervention or placebo group with an allocation ratio of 1:1 using block randomization. Allocation concealment was done by using pre-packed sequentially numbered containers for each patient. All the containers having the study intervention were identical in appearance with similar looking capsules, equal in weight and tamper–proof. Eight bottles (each bottle, 95 capsules) with same code were prepared for each patient for the treatment period and was given sequentially. The investigators, care providers and participants remained blinded to treatment allocation until the outcomes had been analysed. The participants were enrolled, assigned to the intervention and followed up throughout the study by the clinical team.

### Cinnamon and placebo capsules

Raw cinnamon was bought from K.V Spices Private Limited, Delhi [approved by Indian Government, certified with the Food Safety Management System Certificate (International Organization for Standardization 22,000) and National Small Industries Corporation Limited CRISIL Performance and Credit Rating for Small Scale Industries of the company]. The quality control report obtained from the company from where the cinnamon was procured included parameters like moisture, total ash on dry basis, volatile oil content, extraneous matter, and insect damaged matter. Cinnamon was powdered in Pulverizer Spice Grinder machine at 20 °C in humidity less than 50% in order to prevent any loss of active ingredients which could occur due to exposure to high heat. Cinnamon capsules were made using NJP1200 automatic capsule filling machine (Shanghai Develop Machinery Company Limited, China). Each capsule had dark brown color gelatin cover [Health Caps India Limited (World Health Organization and Good Manufacturing Practices (GMP) certified)] and contained 500 mg of cinnamon. The grinding, manufacturing and labeling of cinnamon capsules were done by Basic Human Health Private Limited, New Delhi, India (a GMP certified pharmaceutical company).

The placebo capsules for control subjects consisted of wheat flour as after roasting, the colour imparted was almost similar to cinnamon. Wheat flour has also been used as a placebo along with cinnamon in earlier trials by Khan et al. [18], Vanschoonbeek et al. [26], Blevins et al. [27], and Ziegenfuss et al. [19]. Moreover, since wheat is the staple cereal for Asian Indians, the small amount of wheat (2.5 g/d) given as placebo to the control subjects was not likely to cause major changes in the dietary macronutrient intake. These placebo capsules were made from Aashirwad Whole Wheat Flour (International Organization for Standardization 22,000 certified, followed Good Hygiene Practice and Good Manufacturing Practice) which was dry roasted on low heat till it attained a brownish color similar to that of cinnamon. After cooling the wheat flour, cinnamon essence was added to it [Sonarome Private Limited (International Organization for Standardization 9001 and International Pharmaceutical Excipients Council {IPEC} & Pharmaceutical Quality Group {PQG} GMP certified)]. The composition of the essence was not tested in our labs. However, a material data safety sheet was obtained from the company (Sonarome Private Limited) from where the essence was purchased, which stated the essence contained “natural, nature identical and artificial ingredients”. A very small quantity was used to flavor the entire batch of wheat flour and hence may not exert any potential effect on metabolic syndrome indicators in the placebo group. This mixture was stored in big steel containers which were transported to the same pharmaceutical company for filling into capsules. Placebo capsules were prepared after the batch of cinnamon capsules was packed so that there were no chances of contamination. Capsules similar in color, shape and size to that of cinnamon capsules with dark brown gelatin cover were filled with 416.6 mg of whole wheat flour each.

### Study intervention and compliance

During the run in period (four weeks), all participants were advised to consume diets formulated according to the Dietary Guidelines for Asian Indians [28].These included individualized diet charts (1200 Kcals, 1400 Kcals, 1600 Kcals) and advice on the importance of balanced diet, consuming salad with each meal, decreasing consumption of fried snacks, increasing the intake of fruits and vegetables etc. They were also counseled regarding the importance of physical activity and were motivated to incorporate physical activity in their lifestyle according to guidelines for Asian Indians [29] i.e. by going for a brisk walk for 45 min every day. Each participant participated in at least two interactions during the run-in period to gain detailed knowledge about the study and to ensure compliance. Post randomization, the participants were assigned to either the cinnamon intervention group (3 g/daily) or the placebo group (wheat flour, 2.5 g/daily) receiving the intervention in the form of capsules for 16 weeks. Each subject in both groups was instructed to consume two capsules of their respective supplement after breakfast, lunch and dinner as well as continue with the diet and physical activity recommended to them during run in period. Body mass index (BMI), waist circumference (WC), waist-hip ratio (WHR), percentage body fat, FBG, HbA1c, lipid profile, high-sensitivity C-reactive protein (hs-CRP), SBP, and diastolic blood pressure (DBP) were assessed before and after the intervention.

Two cinnamon or placebo capsule bottles were given every 4 weeks to the participants of the respective groups on their monthly visit to the hospital. Compliance to the protocol was monitored by biweekly checks by telephone calls, SMS and emails. Patients were asked to bring back the empty bottles with the left over capsules (if any) at the time of the monthly visit to the hospital.

### Methods

Weight, height, WC, blood pressure and percent body fat were measured using standard procedures. A fixed stadiometer with movable headboard was used for measuring height. Weight and percent body fat measurements of the subjects were taken by a digital bio electric impedance-based body fat analyser (Tanita Body Composition Analyser, SC300, Japan). Subjects wore minimal clothing and were without shoes and socks while the measurements were taken. In order to measure the blood pressure, the individual was made comfortable and seated in a chair for at least five minutes before the measurement. It was measured by a standard mercury sphygmomanometer (Industrial Electronic and Allied Products, Pune, India) according to JNC guidelines. For measuring waist and hip circumferences, non- stretchable tape was used and measurements done according to the standard guidelines. Lifestyle factors assessed were defined as adequate physical activity (for about 150 min of aerobic exercise per week), chronic smoker (any amount of smoking/chewing tobacco) and alcohol consumption [up to 1 drink (30 ml) per day for women and up to 2 drinks (60 ml) per day for men].

Fasting blood samples (12 ml of blood after an overnight fasting of 12 h) and post prandial samples (collected after 2 h of having breakfast) were analysed. The samples were centrifuged (1700×g; 10 min; 4 °C) immediately after collection and off-the-clot, non-haemolysed serum samples were removed with a micropipette. The serum samples were kept at −32 °C until biochemical analysis (Fasting blood glucose, post prandial glucose and levels of total cholesterol, triglyceride and HDL-c) was performed according to methods described previously [30]. Measurement of hs-CRP was done by using a kit based on the ELISA method (Biocheck, Inc. CA, USA). All the biochemical tests were done at fasting and this practice was consistent for pre and post phase. These test were done at SRL Diagnostic Laboratory (Government of India Certified Laboratory).

### Statistical analysis

Sample size was calculated for a two group parallel double blind randomized control trial. A change in FBG was taken as the primary outcome variable. Assuming the mean ± SD (116.3 ± 12.8 mg/dL) of pre-treatment reported from the earlier human trial [19] for the wheat flour group and anticipating 8% reduction in the intervention arm, the estimated sample size was 40 participants in each arm with level of significance of 0.05 and 80% power. Considering the attrition rate of 30%, 58 participants were enrolled in each arm (116 in total).

The data was entered, managed in an excel spreadsheet and analysed using Stata 9.0 (College Station, Texas, USA). The data were presented as number (%) or mean ± SD / median (min – max) as appropriate. Baseline categorical and continuous characteristics were analysed using chi square test and two sample t test/ Wilcoxon ranksum test respectively. The primary outcome (FBG) and secondary outcomes were analysed using intention to treat analysis. The missing values were replaced using Baseline Observations Carried Forward Method (BOCF). Analysis of covariance method was used to compare the difference in mean values of primary and secondary outcomes adjusting for baseline BMI. The hs-CRP was compared between the groups using Wilcoxon ranksum test since the data was not following the normal distribution. A *p* value <0.05 was considered statistically significant.

## Results

**Fig. 1:**
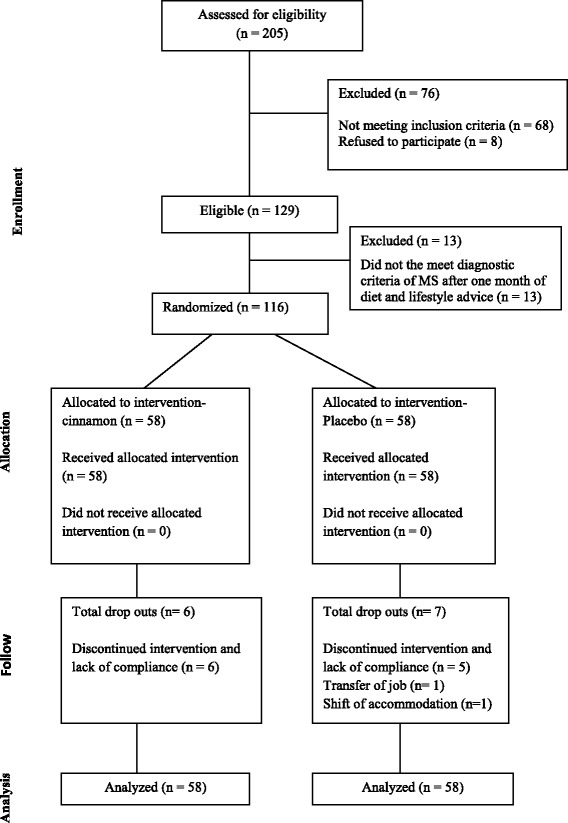
Study flow chart

A total of 129 metabolic syndrome individuals were enrolled in the study at the baseline out of which 116 who met the diagnostic criteria of metabolic syndrome after the run in period of 4 weeks received the allocated study intervention. Thirteen patients (6 in cinnamon and 7 in placebo group) were dropouts (Fig. 1).

There were 64 men and 52 women with a mean age of 44.8 ± 7.8 years and a mean height of 163.6 ± 1.1 cm in the present trial. At baseline, no significant difference was found between the two groups in characteristics except for weight and BMI. The mean weight and BMI (89.1 ± 14.1 kg; 33.6 ± 5.4 kg/m^2^) were higher in the cinnamon group as compared to the placebo group (Table 1).

**Table 1:** Baseline characteristics

Variables	Cinnamon group	Placebo group	*P*- value
	(*n* = 58)	(*n* = 58)	
Age (y)	44.3 ± 7.2	45.1 ± 8.4	0.546
Gender			0.709
Males	31 (53.4)	33 (56.9)	
Females	27 (46.5)	25 (43.1)	
Height (cm)	164.0 ± 8.5	163.3 ± 9.3	0.680
Weight (kg)	89.1 ± 14.1	82.1 ± 14.1	0.009 *
BMI (kg/m^2^)	33.6 ± 5.4	31.2 ± 4.4	0.010*
WC (cm)	106.6 ± 9.2	103.9 ± 9.3	0.118
WHR	0.94 ± 0.07	0.96 ± 0.08	0.293
Body fat (%)	37.9 ± 9.5	36.2 ± 8.4	0.295
SBP (mmHg)	135.8 ± 8.3	135.9 ± 8.6	0.939
DBP (mmHg)	88.0 ± 6.1	88.1 ± 6.1	0.938
Adequate physical activity^a^	13 (22.4)	21 (36.2)	0.103
Chronic smoker^a^	8(13.8)	14(24.1)	0.155
Alcohol consumption^a^	24(41.4)	22(37.9)	0.704

*BMI* body mass index, *DBP* diastolic blood pressure, *SBP* systolic blood pressure, *WC* waist circumference, *WHR* waist hip ratio

*p value <0.05, statistically significant, ^a^See Methods for definitions

Values were presented as n (%) and mean ± SD

The cinnamon intervention group showed a significant decrease in FBG [5.7 ± 0.6 mmol/L (baseline) to 5.2 ± 0.3 mmol/L (16 weeks), *p* = 0.001], HbA1c [43.7 ± 5.4 mmol/mol (baseline) to 39.6 ± 5.01 mmol/mol (16 weeks), *p* = 0.023] and PPG [7.4 ± 1.6 mmol/mol (baseline) to 6.9 ± 1.4 mmol/mol (16 weeks), *p* = 0.030] (Table 2). The cinnamon intervention resulted in a significantly higher decrease in weight, WC, WHR, percentage body fat, total cholesterol, serum triglycerides, LDL-C, LDL: HDL, SBP, DBP and significant increase in HDL-C as compared to the placebo group (Tables 2 and 3).

**Table 2:** Changes in body composition and blood pressure after 16 weeks of intervention

Body composition and blood pressure	Cinnamon group (*n* = 58)	Placebo group (*n* = 58)	Difference between Means (95% CI)	*P* value †
Weight (kg)^a^				
Baseline	89.1 ± 14.1	82.1 ± 14.1	7.0 (1.7, 12.1)	0.009*
16 weeks				
Unadjusted	85.6 ± 13.7	81.7 ± 14.6	−3.9 (− 9.1, 1.3)	0.148
Adjusted			3.0 (−2.3, 3.8) ‡	0.001*
BMI (kg/m^2^)				
Baseline	33.6 ± 5.4	31.2 ± 4.4	−2.4 (−0.5, − 4.2)	0.010*
16 weeks				
Unadjusted	32.3 ± 5.2	31.0 ± 4.4	−1.3 (− 3.1, 0.5)	0.162
Adjusted			1.3 (0.9, 1.5) ‡	0.001*
WC (cm)				
Baseline	106.6 ± 9.2	103.9 ± 9.3	−2.7 (−6.1, 0.7)	0.118
16 weeks				
Unadjusted	101 ± 9.1	103.1 ± 9.7	2.2 (−1.3, 5.6)	0.223
Adjusted			4.8 (1.9, 7.7) ‡	0.002*
WHR				
Baseline	0.94 ± 0.07	0.96 ± 0.08	0.02 (0.01, 0.04)	0.293
16 weeks				
Unadjusted	0.91 ± 0.07	0.96 ± 0.08	0.05 (0.01, 0.06)	0.005*
Adjusted			0.03 (0.004, 0.06) ‡	0.028*
Body fat percentage				
Baseline	37.9 ± 9.5	36.2 ± 8.4	- 1.7 (−5.1, 1.5)	0.295
16 weeks				
Unadjusted	36.4 ± 9.5	36.2 ± 8.3	- 0.2 (−3.4,3.1)	0.928
Adjusted			3.0 (0.7, 5.3) ‡	0.011*
SBP (mmHg)				
Baseline	135.8 ± 8.3	135.9 ± 8.6	−0.1 (− 3.1, 3.2)	0.939
16 weeks				
Unadjusted	122.2 ± 6.3	130.5 ± 10.5	8.3 (5.1, 11.4)	0.001*
Adjusted			8.3 (5.0, 11.6) ‡	0.001*
DBP (mmHg)				
Baseline	88.0 ± 6.1	88.1 ± 6.1	0.1 (− 2.1, 2.3)	0.938
16 weeks				
Unadjusted	79.9 ± 5.7	86.9 ± 6.2	7.0 (4.8, 9.2)	0.001*
Adjusted			6.9 (4.6, 9.1) ‡	0.001*

BMI, body mass index; CI, confidence interval; DBP, diastolic blood pressure; SBP, systolic blood pressure; WC, waist circumference; WHR, waist hip ratio

^†^Intervention vs. placebo

^‡^Difference between placebo and cinnamon groups adjusted for BMI and a adjusted for baseline weight except weight using analysis of covariance

^§^Values were presented as mean ± SD and median (min - max)

^*^ *p* value <0.05

**Table 3:** Changes in biochemical parameters after 16 weeks of intervention

Biochemical parameters	Cinnamon group (*n* = 58)	Placebo group (*n* = 58)	Difference between Means (95% CI)	*P* value ^a^
Intention to treat analysis				
FBG (mmol/L)				
Baseline	5.7 ± 0.6	5.6 ± 0.5	−0.1(− 0.2, 0.1)	0.485
16 weeks				
Unadjusted	5.2 ± 0.3	5.5 ± 0.4	0.3 (0.1, 0.4)	0.001*
Adjusted			0.3 (0.2, 0.5) ^b^	0.001*
Per protocol analysis				
FBG (mmol/L)				
	(*n* = 52)	(*n* = 51)		
Baseline	5.7 ± 0.6	5.6 ± 0.5	−0.1 (− 0.3, 0.1)	0.528
16 weeks				
Unadjusted	5.2 ± 0.3	5.5 ± 0.4	0.3 (0.1, 0.4)	0.001*
Adjusted			0.4 (0.2, 0.5) ^b^	0.001*
Intention to treat analysis				
HbA1c (mmol/mol)				
Baseline	43.7 ± 5.4	42.4 ± 6.07	−1.3 (−3.3, 0.8)	0.250
16 weeks				
Unadjusted	39.6 ± 5.01	42.5 ± 6.8	2.9 (0.6, 5.08)	0.011*
Adjusted			2.6 (0.4, 4.9) ^b^	0.023*
PPG (mmol/L)				
Baseline	7.4 ± 1.6	7.3 ± 1.4	−0.1 (−0.6, 0.4)	0.727
16 weeks				
Unadjusted	6.9 ± 1.4	7.4 ± 1.4	0.5 (−0.01, 1.06)	0.055*
Adjusted			0.6 (0.1, 1.2) ^b^	0.030*
Total cholesterol (mmol/L)				
Baseline	5.23 ± 0.71	5.05 ± 0.94	−0.17 (− 0.48, 0.13)	0.259
16 weeks				
Unadjusted	4.68 ± 0.64	5.09 ± 0.92	0.41 (0.10, 0.69)	0.007*
Adjusted			0.42 (0.12, 0.73) ^b^	0.006*
Serum Triglycerides (mmol/L)				
Baseline	1.97 ± 0.44	1.91 ± 0.48	−0.06 (−0.23, 0.10)	0.468
16 weeks				
Unadjusted	1.65 ± 0.39	1.93 ± 0.47	0.28 (0.11, 0.43)	0.001*
Adjusted			0.20 (0.05, 0.35) ^b^	0.010*
HDL (mmol/L)				
Baseline	0.97 ± 0.15	0.96 ± 0.23	−0.003 (−0.07, 0.06)	0.922
16 weeks				
Unadjusted	1.02 ± 0.15	0.94 ± 0.22	0.08 (0.01, 0.15)	0.024*
Adjusted			0.08 (0.005, 0.15) ^b^	0.035*
LDL (mmol/L)				
Baseline	3.46 ± 0.60	3.29 ± 0.68	- 0.16 (−0.40, 0.07)	0.178
16 weeks				
Unadjusted	3.01 ± 0.58	3.33 ± 0.66	0.32 (0.09, 0.55)	0.006*
Adjusted			0.37 (0.13, 0.60) ^b^	0.003*
LDL: HDL				
Baseline	3.62 ± 0.79	3.55 ± 0.98	- 0.07 (−0.40, 0.25)	0.641
16 weeks				
Unadjusted	2.97 ± 0.68	3.66 ± 0.98	0.69 (0.37,0.99)	0.001*
Adjusted			0.72 (0.40, 1.04) ^b^	0.001*
hs- CRP^c^				
Baseline	2.8 (2.33–3.30)	2.4 (1.98–2.86)	-	0.255
16 weeks	2.5 (2.03–2.94)	2.6 (2.21–3.09)	-	0.382

*CI* confidence interval, *FBG* fasting blood glucose, *HbA1c* glycosylated haemoglobin, *HDL-C* high-density lipoprotein cholesterol, *LDL–C* low-density lipoprotein cholesterol, *LDL: HDL* low-density lipoprotein cholesterol high-density lipoprotein cholesterol ratio, *PPG* postprandial blood sugar

^a^ Intervention versus placebo

^b^ Difference between placebo and cinnamon groups adjusted for BMI using analysis of covariance

^c^Values depicted as geometric mean (95%CI)

*p value <0.05

**Fig. 2:**
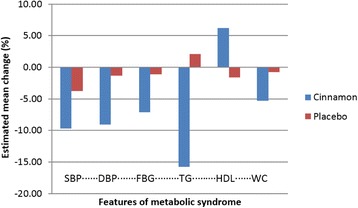
The bar diagram showing the estimated mean change in percentage of individual components of metabolic syndrome. DBP, diastolic blood pressure; FBG, fasting blood glucose; HDL-C, high-density lipoprotein cholesterol; SBP, systolic blood pressure; WC, waist circumference; TG, serum triglyceride

The estimated mean percent change in various components of metabolic syndrome after intervention of 16 weeks as compared to placebo is shown in Fig. 2. There was a higher decrease in the mean systolic blood pressure (*p* < 0.001), diastolic blood pressure (*p* < 0.001), fasting blood glucose (*p* < 0.001), triglycerides (*p* < 0.001), and waist circumference (*p* < 0.001) and a greater increase in high density lipoprotein (*p* < 0.001) in the cinnamon group than the placebo group. The presence of characteristic features of metabolic syndrome was significantly reduced in the cinnamon intervention group (34.5%) vs. placebo group (5.2%). No harmful effects were reported on consumption of cinnamon or placebo capsules.

## Discussion

This study shows a considerable beneficial effect of cinnamon supplementation on Asian Indians with metabolic syndrome as evident from a significant decrease in hyperglycemia, body weight, total adiposity, abdominal adiposity and serum lipid levels with the use of 3 g/day of cinnamon as compared to placebo over a period of 16 weeks. A noteworthy finding in the present investigation is a significant increase in serum HDL-C levels upon intervention with cinnamon; an observation which has not been reported previously.

Previous studies report beneficial effects of cinnamon on normal subjects, and those with prediabetes, T2DM and polycystic ovarian disease (PCOD). In 14 healthy subjects with BMI 22.6 ± 2.2 kg/m^2^, Hlebowicz et al. [19] showed reduced post prandial blood glucose levels and decreased gastric emptying rate by ingestion of 6 g cinnamon daily in rice pudding. On the contrary, Tang and colleagues reported no significant effect on urinary oxalate excretion, plasma lipids and plasma glucose levels of 11 healthy subjects with the use of 3 g of cinnamon daily for a period of four weeks [31]. Another study by Hlebowicz et al. [32], in 15 healthy subjects showed reduced levels of postprandial insulin and increased glucagon-like peptide-1 (GLP-1) levels with the use of cinnamon. Multiple effects of cinnamon via decrease gastric emptying, reduced insulin levels and increase GLP-1 [32] may explain the overall improvement in glycemic parameters and to some extent weight loss in the present study.

A number of intervention studies have been done on patients with T2DM using cinnamon. Khan et al. [18] demonstrated a decrease in FBG and lipids in 60 patients with T2DM. In this study, three different doses of cinnamon (1, 3 and 6 g/day) were given to the subjects for a period of 40 days, however similar biochemical effects were seen. Importantly, the beneficial effects were also seen 20 days after the washout phase. Significant reductions in serum total cholesterol, triglycerides, LDL- C were also documented in present study, however, the magnitude of change were slightly lesser in comparison to those reported by Khan et al. [18]. This could be explained by the fact that Khan et al. [18] performed the study on poorly controlled diabetic patients and severely dyslipidemic individuals, mostly on treatment, whereas in the present investigation the recruited sample consisted of treatment naïve individuals with metabolic syndrome having slightly or moderately high levels of lipids. Studies by Vanschoonbeek et al. [26] and by Blevins et al. [27] however, showed no significant effect of cinnamon supplementation on patients with T2DM. However, in these studies, a low dose (1–1.5 g) of cinnamon was used, and subjects were also on other anti-hyperglycemic medications. All these studies were placebo control trials where similar to our trial, wheat flour was used as placebo.

Very few studies have been conducted on individuals without diabetes having a dysmetabolic state, and most of these studies involved small number of subjects. In a pilot study by Solomon and Blanin [16], increase in insulin sensitivity with cinnamon extract (1 g/day) was seen in a limited number of subjects (*n* = 15) with PCOD. In a placebo controlled trial, 22 subjects with impaired fasting glucose were given cinnamon extract of 500 mg/day for a period of 12 weeks. This resulted in improvements in fasting blood glucose and antioxidant levels [33]. Similarly, Ziegenfuss et al. [19] researched 22 individuals with prediabetes water soluble cinnamon extract (500 mg/day) intervention for 12 weeks, and showed decreased FBG (8.4%), SBP (3.8%) and improved body composition (increase in lean mass by 1.1% and decrease in body fat by 0.7%) [19]. While the study of Ziegenfuss et al. [19] shows decrease of FBG to be slightly higher in the cinnamon group as compared to our results, it may be attributed to slightly higher baseline values of FBG in cinnamon group in the previous study.

Anderson et al. [15] demonstrated that the in vitro insulin-potentiating activity found in cinnamon is present in the aqueous fraction. The water-soluble cinnamon polyphenol compounds that display insulin-potentiating, antioxidant, and related activities are Type A doubly linked procyanidin oligomers of the catechins and epicatechin. Further methyl chalcone polymer (MHCP) in cinnamon enhances the triacylglycerol lipase activity that hydrolyzes dietary fat, increases glycogen synthesis in liver, enhances glucose uptake and phosphorylation of insulin receptor in skeletal muscles and adipocytes [14].

The possible mechanism of action of cinnamon may involve inhibition of activity of enzymes involved in carbohydrate metabolism (pancreatic α–amylase and α–glucosidase) [34–36], stimulation of cellular glucose uptake by membrane translocation of GLUT-4 [37, 38], stimulation of glucose metabolism and glycogen synthesis, inhibition of gluconeogenesis by effects on key regulatory enzymes [38, 39] and stimulation insulin release and potentiating insulin receptor activity [37, 39, 40]. In animal studies cinnamon has been shown to have lipid lowering properties. Cinnamate, a phenolic compound, found in the inner bark of cinnamon, lowers cholesterol level in high fat fed rats by inhibiting hepatic HMG Co-A reductase activity [41]. Further, it also suppresses lipid peroxidation via enhancement of hepatic antioxidant enzyme activity [42]. Also, in a clinical trial of cinnamon (1, 3, or 6 g/day) vs. placebo, given to patients with diabetes for 40 days with a 20-day washout period, these doses of cinnamon led to similar reduction in the following parameters; fasting glucose by 18–29%, triglycerides 23–29%, and cholesterol 12–26% [18]. Nyadjeu et al. [43, 44] have also showed a blood pressure lowering effect of cinnamon in rat models. The aqueous extract of cinnamon stem bark has been shown to reduce sucrose-induced elevation in systolic blood pressure of spontaneously hypertensive rats [45]. In humans, a placebo controlled trial on prediabetic individuals wherein a dose of 500 mg of water soluble cinnamon extract was administered for a period of 12 weeks, showed a decrease of 3.8% in systolic blood pressure [19]. In a meta-analysis on the effect of short-term administration of cinnamon on blood pressure regulation in prediabetes and type 2 diabetes, Aklien et al. [46] concluded that cinnamon significantly decreased systolic blood pressure and diastolic blood pressure by 5.3 mmHg (95% Cl, −6.89 to −3.89 mmHg) and 2.6 mmHg (95% CI, −4.53 to −0.66 mmHg) in patients with type 2 diabetes and pre-diabetic individuals. Moreover, how cinnamon could decrease adiposity has been researched and debated. Several mechanisms could be operative; inhibition of differentiation of adipocyte, effects on intestinal lipid absorption, induction of fatty acid oxidation and antagonism at cannabinoid receptors [47].

Indeed, if cinnamon acts at the cellular level in improving insulin resistance, it could be of great value to Asian Indians who have high predisposition for insulin resistance, more than other ethnic groups [48]. In addition, decrease in overall obesity and abdominal obesity would be of relevance for Asian Indians, since much of their tendency of development of metabolic syndrome and diabetes could be ascribed to such body composition characteristics [5]. Further the cost of the cinnamon capsules in the given dosage (6 capsules/daily) for one patient is $ 0.03 which is cost effective in comparison to anti-hyperglycemic or weight loss medications. Cinnamon is also used as a flavoring agent in Indian cooking and hence it is easy to incorporate in the daily diets.

There are a number of strengths of our study. The study has been robustly designed as double blind randomized placebo control trial. Further, strict quality control has been maintained in manufacture of cinnamon and wheat flour capsules. Finally, in our study, cinnamon appears to be reasonably safe at the dose and duration studied.

There are a few limitations in our study. Firstly, the various parameters could be again measured at midpoint for further comparisons. Also, further investigations could be done with varying doses of cinnamon as well as on a larger sample for a longer duration. It may be worthwhile to also investigate the biomarkers of cinnamon intake and its effect of on other markers of insulin resistance (insulin and adiponectin levels). Moreover, effects of cinnamon on beta cell function and glucagon levels should be assessed. Additionally, all the participants in our study were obese, we cannot generalize this result to the lean population. Finally, this research was conducted on a limited sample. To further substantiate the benefits of oral cinnamon, the research should be conducted on a larger sample.

## Conclusion

In this 16 weeks study, we show significant decrease in measures of glycemia, adiposity including abdominal obesity, lipids, blood pressure and major decrease in percentage of individuals having metabolic syndrome with single nutrient intervention of cinnamon. While the results of this investigation are promising, they should be tested in a larger sample over longer period of time.

## Abbreviations

BMI: Body mass index
CVD: Cardiovascular disease
DBP: Diastolic blood pressure
FBG: Fasting blood glucose
GLP-1: Glucagon-like peptide-1
HbA1C: Glycosylated haemoglobin
HDL-C: High density lipoprotein-cholesterol
hs- CRP: High-sensitivity C-reactive protein
IPEC: International Pharmaceutical Excipients Council
LDL-C: low density lipoprotein-cholesterol
MHCP: Methyl chalcone polymer
PCOD: Polycystic ovarian disease
PPG: Postprandial blood glucose levels
PQG: Pharmaceutical Quality Group
SBP: Systolic blood pressure
T2DM: Type 2 diabetes
WC: Waist circumference
WHR: Waist-hip ratio
